# Tumor necrosis factor inhibitors and cardiovascular outcomes in adults with moderate-to-severe plaque psoriasis: a systematic review and meta-analysis^☆^

**DOI:** 10.1016/j.abd.2026.501473

**Published:** 2026-10-27

**Authors:** Luisa Fernanda Mesa Rodríguez, Alejandro Zambrano Aristizábal, Juan David Vega Padilla

**Affiliations:** a Department of Epidemiology, Universidad El Bosque, Bogotá, Colombia; b School of Medicine, Fundación Universitaria de Ciencias de la Salud, Bogotá, Colombia; c Department of Epidemiology, Universidad de Boyacá, Tunja, Colombia

**Keywords:** Adalimumab, Cardiovascular diseases, Etanercept, Heart failure, Myocardial infarction, Psoriasis, Stroke

## Abstract

**Background:**

Moderate-to-severe plaque psoriasis is associated with persistent systemic inflammation and increased cardiovascular risk.

**Objectives:**

To evaluate the association between anti-TNF therapy and clinical cardiovascular outcomes in adults with moderate-to-severe plaque psoriasis.

**Methods:**

The authors conducted a systematic review and meta-analysis in accordance with PRISMA 2020 and prospectively registered the protocol in PROSPERO (CRD420251270784). Randomized controlled trials and comparative observational studies evaluating anti-TNF therapy versus placebo, no anti-TNF exposure, topical therapy and/or phototherapy, or non-biologic systemic therapy were eligible. Outcomes of interest were Major Adverse Cardiovascular Events (MACE), myocardial infarction, stroke, cardiovascular death, heart failure, atrial fibrillation, venous thromboembolism, and all-cause mortality.

**Results:**

Fourteen studies were included. In randomized evidence, anti-TNF therapy was not associated with significant differences in the risk of MACE, myocardial infarction, stroke, or heart failure. In observational analyses stratified by comparator, anti-TNF therapy was associated with a lower pooled risk of reported cardiovascular events versus topical therapy, but not versus oral therapy or phototherapy.

**Study limitations:**

The available evidence was limited by short follow-up in randomized trials, low cardiovascular event counts, wide confidence intervals, and indirect assessment of cardiovascular outcomes, as most trials were designed primarily for dermatologic efficacy and general safety rather than cardiovascular endpoints.

**Conclusions:**

Anti-TNF agents were not associated with a clear increase in major cardiovascular risk and appear to have an acceptable short-term cardiovascular safety profile. However, current evidence does not establish a conclusive cardioprotective effect, and larger prospective studies with longer follow-up are needed.

## Introduction

Psoriasis is a chronic immune-mediated inflammatory disease with systemic manifestations beyond the skin.^1^ It is associated with an increased risk of cardiovascular disease and adverse atherosclerotic outcomes.^2^ This risk appears to increase with disease severity, and systemic inflammation has been proposed as a key biological mechanism underlying this association.^3^ In patients with moderate-to-severe psoriasis, anti-TNF agents remain one of the biologic options used in systemic treatment.^1^, ^4^ Because of their systemic anti-inflammatory effects, these drugs have been proposed as potential modifiers of cardiovascular risk beyond skin clearance.^5^ However, recent evidence on the cardiovascular effects of systemic therapies for psoriasis remains heterogeneous.^6^ A recent systematic review and meta-analysis in immune-mediated inflammatory diseases reported a lower risk of MACE with TNF inhibitors compared with conventional systemic non-biologic therapies, including in the psoriasis/psoriatic arthritis subgroup.^7^ While some population-based studies have reported lower rates of cardiovascular disease among patients receiving biologics, including anti-TNF agents, other analyses have shown inconsistent signals according to the comparator and study design.^6^, ^8^ In this context, a focused synthesis of the evidence on the association between anti-TNF use and clinical cardiovascular outcomes in adults with moderate-to-severe plaque psoriasis is justified.^2^, ^8^ The objective of this systematic review was to evaluate the association between anti-TNF use and the risk of clinical cardiovascular outcomes in adults with moderate-to-severe plaque psoriasis.

## Methods

### Study design

This systematic review and meta-analysis were conducted in accordance with the PRISMA 2020 statement and was prospectively registered in PROSPERO under the identifier CRD420251270784.

### Eligibility criteria

The authors included human studies enrolling participants aged 18-years or older with moderate-to-severe plaque psoriasis, according to each study definition. Eligible designs were randomized controlled trials and comparative observational studies, including prospective or retrospective cohorts and case-control studies. The exposure of interest was anti-TNF therapy for plaque psoriasis in adults, including adalimumab, etanercept, infliximab, and certolizumab pegol, including biosimilars. Eligible comparators were placebo, no anti-TNF exposure, topical therapies and/or phototherapy, and non-biologic systemic therapies, provided that anti-TNF exposure was not included during the risk period. The authors excluded studies without a comparator group, pediatric studies, studies focused exclusively on psoriasis subtypes other than plaque psoriasis without extractable data for plaque psoriasis, mixed cohorts with psoriatic arthritis or other immune-mediated diseases without psoriasis-specific estimates, studies in which anti-TNF exposure could not be separated from other biologic classes, narrative reviews, editorials, comments, conference abstracts without sufficient information, and designs not suitable for the assessment of incident cardiovascular events. For synthesis, studies were grouped according to study design, outcome, and comparator category.

### Information sources and search strategy

The search was conducted in CENTRAL, PubMed/MEDLINE, Scopus, Web of Science, and Ovid, without language or publication-date restrictions. Additional studies were sought through citation searching and manual screening of other relevant sources. The search strategies combined terms for plaque psoriasis, anti-TNF therapy, clinically relevant cardiovascular outcomes, and comparative study designs. The search strategy for PubMed was as follows: (((("plaque psoriasis"[tiab] OR "chronic plaque psoriasis"[tiab] OR "plaque-type psoriasis"[tiab] OR "plaque type psoriasis"[tiab] OR "psoriasis vulgaris"[tiab] OR "vulgaris psoriasis"[tiab] OR "plaque psoriasis vulgaris"[tiab] OR "psoriasis"[tiab])) AND ((anti-tnf[tiab] OR "anti tnf"[tiab] OR "TNF inhibitor*"[tiab] OR "tumor necrosis factor inhibitor*"[tiab] OR "tumour necrosis factor inhibitor*"[tiab] OR adalimumab[tiab] OR etanercept[tiab] OR infliximab[tiab] OR certolizumab[tiab] OR "certolizumab pegol"[tiab]))) AND (("major adverse cardiovascular event*"[tiab] OR MACE[tiab] OR "cardiovascular event*"[tiab] OR "cardiovascular outcome*"[tiab] OR "cardiovascular death"[tiab] OR "cardiovascular mortality"[tiab] OR "myocardial infarction"[tiab] OR "acute coronary syndrome"[tiab] OR stroke[tiab] OR "cerebrovascular accident"[tiab] OR "ischemic stroke"[tiab] OR "haemorrhagic stroke"[tiab] OR "hemorrhagic stroke"[tiab] OR "heart failure"[tiab] OR "venous thromboembolism"[tiab] OR VTE[tiab] OR "deep vein thrombosis"[tiab] OR DVT[tiab] OR "pulmonary embolism"[tiab] OR PE[tiab] OR "atrial fibrillation"[tiab]))) AND ((randomi*[tiab] OR trial[tiab] OR "controlled trial"[tiab] OR cohort[tiab] OR "cohort study"[tiab] OR "cohort studies"[tiab] OR prospective[tiab] OR retrospective[tiab] OR observational[tiab] OR "observational study"[tiab] OR registry[tiab] OR registries[tiab] OR "population-based"[tiab] OR "real-world"[tiab] OR "claims"[tiab] OR "administrative database"[tiab] OR "healthcare database"[tiab] OR "electronic health record*"[tiab] OR EHR[tiab] OR "case-control"[tiab] OR "case control"[tiab] OR "nested case-control"[tiab] OR "nested case control"[tiab] OR "propensity score"[tiab] OR "inverse probability"[tiab] OR IPTW[tiab])). This strategy was adapted for each database. The last search date across all databases was January 25, 2026.

### Study selection process

Title/abstract screening and full-text review were performed independently by the three reviewers, with disagreements resolved by consensus. Study selection followed the sequential stages of identification, screening, eligibility assessment, and inclusion. Reasons for exclusion at the full-text stage were documented explicitly.

### Data extraction and variables

Data extraction was performed independently by the three reviewers, with disagreements resolved by consensus. The authors did not plan to contact study authors to request additional information. Extracted variables included author, year of publication, country, study design, data source, sample size, population characteristics, inclusion and exclusion criteria, definition of anti-TNF exposure, comparator, follow-up duration, and cardiovascular outcomes. Primary outcomes included MACE, preferably defined as a composite of non-fatal myocardial infarction, non-fatal stroke, and cardiovascular death, as well as myocardial infarction, stroke, and cardiovascular death individually. Secondary outcomes were all-cause mortality, heart failure, atrial fibrillation, and venous thromboembolism. Available effect estimates and event counts were also extracted when reported.

### Risk of bias assessment

Risk of bias in randomized controlled trials was assessed using the Cochrane RoB2 tool,^9^ whereas observational studies were evaluated with ROBINS-I.^10^ Assessment was carried out independently by the three reviewers, with discrepancies resolved by consensus.

### Effect measures and synthesis methods

The effect measure used for the meta-analyses was the Risk Ratio (RR) with 95% Confidence Intervals (95% CIs). RRs were selected as the primary summary measure because the randomized trials primarily reported cardiovascular events as safety outcomes with available arm-level event counts, and several studies did not report Hazard Ratios (HRs) or sufficient time-to-event information to permit consistent extraction or pooling of HRs across outcomes. Although HRs are methodologically preferable for time-to-event outcomes because they incorporate the timing of events and varying follow-up duration, their inconsistent availability across the included studies would have substantially reduced the number of studies eligible for quantitative synthesis. Therefore, RRs based on extracted event counts were used to maintain a consistent effect measure across randomized and observational analyses. When adjusted HRs were reported by observational studies, these estimates were extracted and considered narratively in the interpretation of individual studies, but they were not pooled together with RRs to avoid mixing non-equivalent effect measures. Studies were considered eligible for quantitative synthesis when they were clinically comparable in terms of population, exposure, comparator, and outcome. Randomized trials and observational studies were synthesized separately. In observational studies, analyses were further stratified by comparator category: anti-TNF versus topical therapy and anti-TNF versus oral therapy or phototherapy. When quantitative synthesis was not possible, findings were summarized narratively and in descriptive tables. Statistical heterogeneity was assessed using the *I*^2^ statistic. For sparse cardiovascular outcomes, the meta-analyses were performed using the event data reported by the original studies and pooled as RRs with 95% CIs using the Mantel-Haenszel fixed-effect method. This approach was selected because the available data consisted primarily of arm-level event counts, statistical heterogeneity was low or absent across analyses, with *I*^2^ values below 50%, and the method allows a consistent synthesis of dichotomous outcomes across randomized and observational comparisons. Studies reporting zero events in both treatment arms for a given outcome were retained in the qualitative description of safety findings but did not contribute to the pooled relative-effect estimate, as no comparative risk ratio can be estimated from double-zero event data. The applicability of sparse-data methods such as Peto odds ratios or rare-event models was considered; however, these were not selected as the primary approach because several included trials had unequal allocation ratios, heterogeneous follow-up periods, and very limited outcome-specific event counts. In this context, the Mantel-Haenszel fixed-effect method was considered more appropriate for maintaining consistency with the prespecified RR-based synthesis, the low statistical heterogeneity observed across analyses, and the use of arm-level dichotomous event data, while avoiding the assumptions required by Peto’s method. Therefore, rare-event analyses were interpreted cautiously, emphasizing confidence-interval width and consistency across sensitivity analyses rather than statistical significance alone. Sensitivity analyses were performed by sequentially excluding individual studies to assess the robustness of the findings and, when applicable, according to treatment type.

### Publication bias and certainty of the evidence

Given the number of studies included in each quantitative synthesis (fewer than 10 studies per meta-analysis), a formal assessment of publication bias was not feasible. Certainty of the evidence for each outcome was evaluated using the GRADE approach.

## Results

The search identified 1,284 records across databases (CENTRAL = 92; PubMed/MEDLINE = 82; Scopus = 783; Web of Science = 117; Ovid = 210). After removal of 406 duplicates, 878 records underwent title/abstract screening, and 842 were excluded. Subsequently, 36 full-text articles retrieved from the databases were assessed for eligibility, of which 22 were excluded after full-text review. In addition, 10 records identified through other sources were assessed and excluded. Detailed reasons for exclusion are provided in Supplementary Table 1. Finally, 14 studies were included in the systematic review.^11^, ^12^, ^13^, ^14^, ^15^, ^16^, ^17^, ^18^, ^19^, ^20^, ^21^, ^22^, ^23^, ^24^ Of these, 5 studies were included in the meta-analysis of MACE in clinical trials,^12^, ^13^, ^14^, ^15^, ^20^ 3 in the meta-analysis of myocardial infarction in clinical trials,^12^, ^13^, ^15^ 3 in the meta-analysis of stroke in clinical trials,^12^, ^14^, ^20^ 3 in the meta-analysis of heart failure in clinical trials,^11^, ^15^, ^17^ 2 in the observational analysis comparing anti-TNF versus topical treatment,^23^, ^24^ and 4 in the observational analysis comparing anti-TNF versus oral treatment/phototherapy^12^, ^13^, ^14^, ^15^, ^16^, ^17^, ^18^, ^19^, ^20^, ^21^, ^22^, ^23^, ^24^ (Fig. 1).

**Figure 1:**
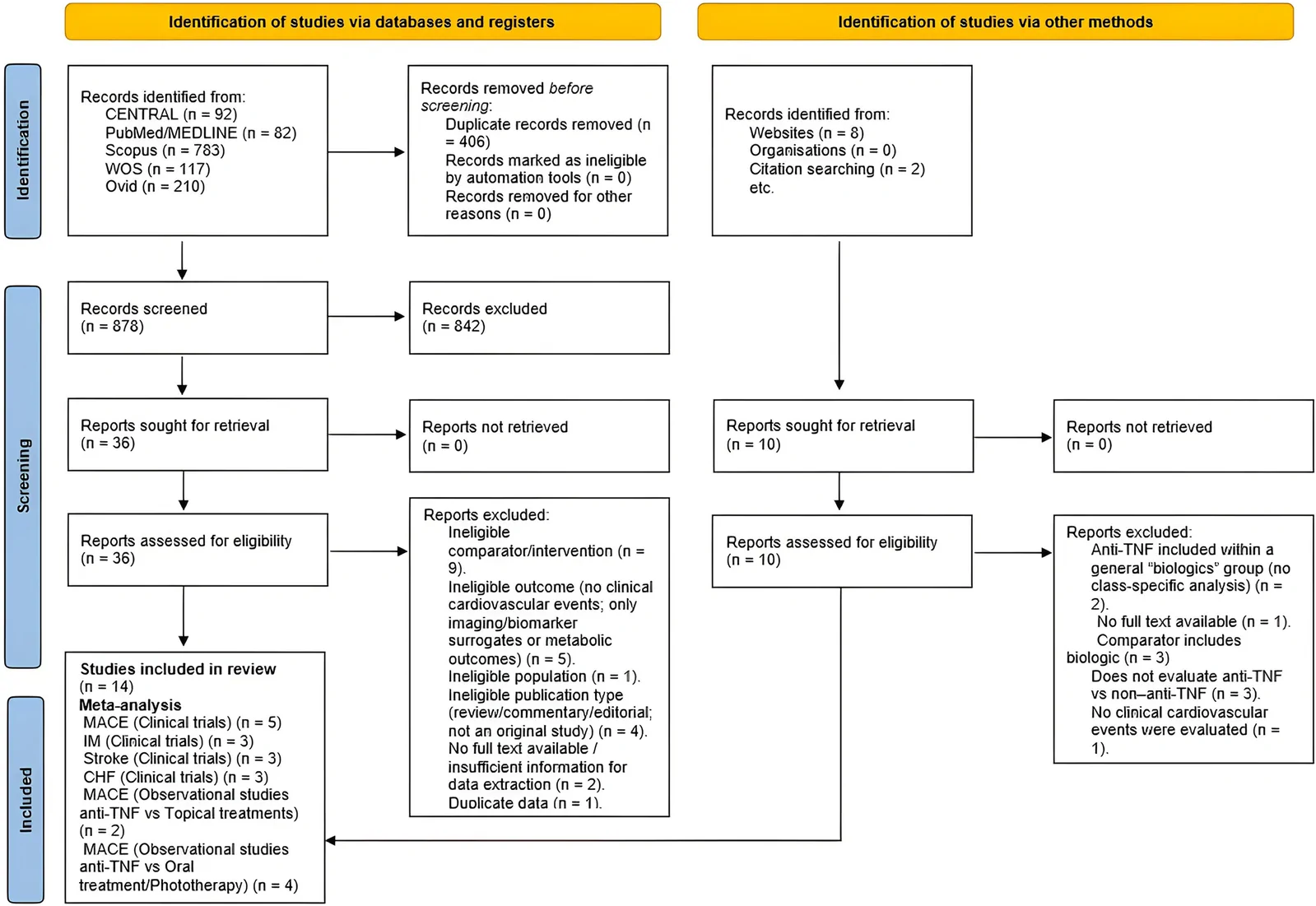
PRISMA 2020 flow diagram for identification, screening, eligibility assessment, and inclusion of studies.

### Qualitative review

#### General characteristics of the included studies

Overall, Table 1 summarizes 14 studies,^11^, ^12^, ^13^, ^14^, ^15^, ^16^, ^17^, ^18^, ^19^, ^20^, ^21^, ^22^, ^23^, ^24^ of which 10 were clinical trials^11^, ^12^, ^13^, ^14^, ^15^, ^16^, ^17^, ^18^, ^19^, ^20^ and 4 were observational studies.^12^, ^13^, ^14^, ^15^, ^16^, ^17^, ^18^, ^19^, ^20^, ^21^, ^22^, ^23^, ^24^ Twelve studies were multicenter.^11^, ^12^, ^13^, ^14^, ^15^, ^16^, ^17^, ^18^, ^19^, ^20^, ^21^, ^22^ The United States contributed the largest number of studies,^11^, ^12^, ^13^, ^14^, ^15^, ^16^, ^17^, ^18^, ^19^, ^20^ followed by Canada,^11^, ^15^, ^17^, ^18^ whereas the United Kingdom and Ireland contributed the main observational studies.^21^, ^22^ In the clinical trials, data sources were derived from multicenter hospital-based, dermatology, academic, and outpatient settings.^11^, ^12^, ^13^, ^14^, ^15^, ^16^, ^17^, ^18^, ^19^, ^20^ In the observational studies, data sources included two cohorts from the prospective BADBIR registry,^21^, ^22^ one study based on hospital electronic health records,^23^ and one integrated population-based database from Kaiser Permanente Southern California.^24^ The clinical trials mainly enrolled adults with moderate-to-severe chronic plaque psoriasis, usually with a minimum disease duration of 6-months and objective severity criteria such as PASI, BSA, and PGA/IGA. In several studies, patients were also required to be candidates for systemic therapy or phototherapy, to have failed or been intolerant to previous treatments, or to be biologic-naïve.^11^, ^12^, ^13^, ^14^, ^15^, ^16^, ^17^, ^18^, ^19^, ^20^ Some trials restricted inclusion to specific clinical phenotypes, such as nail psoriasis or palmoplantar psoriasis.^11^, ^15^ Regarding exclusion criteria, the clinical trials excluded patients with prior exposure to adalimumab or anti-TNF agents, active tuberculosis or tuberculosis without prophylaxis, recent or active malignancy, active infections, relevant autoimmune comorbidities, or concomitant use of treatments such as systemic corticosteroids.^11^, ^13^, ^15^, ^16^, ^17^, ^20^ By contrast, the observational studies included patients identified from prospective registries, electronic health records, or administrative databases, with diagnosis confirmed by a dermatologist or by diagnostic codes, and with operational definitions of exposure based on initiation, switching, or continued use of anti-TNF therapy over a defined minimum period.^12^, ^13^, ^14^, ^15^, ^16^, ^17^, ^18^, ^19^, ^20^, ^21^, ^22^, ^23^, ^24^ In these studies, exclusion criteria generally included patients with previous myocardial infarction or prior major adverse cardiovascular events, although in some registries selection depended mainly on the type of treatment received.^12^, ^13^, ^14^, ^15^, ^16^, ^17^, ^18^, ^19^, ^20^, ^21^, ^22^, ^23^, ^24^

**Table 1:** Characteristics of the included studies.^11^, ^12^, ^13^, ^14^, ^15^, ^16^, ^17^, ^18^, ^19^, ^20^, ^21^, ^22^, ^23^, ^24^

Part A. Clinical trials											
Study	Country / source	Design	Population	Exclusion criteria	Total N	Anti-TNF	Comparator	Follow-up	CV outcomes	CV results	Main finding
Elewski et al., 2018 ^11^	Multinational; 32 dermatology centers and hospitals	Phase III, randomized, double-blind, placebo-controlled	Adults with moderate-to-severe plaque psoriasis and nail involvement; duration ≥ 6 months; severity criteria based on BSA, mNAPSI, and PGA.	Previous adalimumab exposure, use of systemic corticosteroids, or concomitant treatment for nail psoriasis.	217 (109 / 108)	Adalimumab SC: 80 mg initial dose; then 40 mg every 2 weeks.	Placebo	26 weeks	Serious CV events such as AF, carotid stenosis, and heart failure; no formal MACE definition.	AF: 1 case in placebo. Congestive heart failure: 1 case in adalimumab. No MI, stroke, or CV death.	CV events were infrequent and reported only as serious adverse events.
Cai et al., 2017^12^	China; 16 hospital centers	Phase III, randomized, double-blind, placebo-controlled	Adults with stable plaque psoriasis for at least 6 months, moderate-to-severe disease, and lack of response or intolerance to previous systemic therapy.	Previous biologic exposure and use of systemic psoriasis therapies within the previous 28 days.	425 (338 / 87)	Adalimumab SC: 80 mg initial dose; then 40 mg every 2 weeks.	Placebo	24 weeks (12 placebo-controlled)	MI, cerebral infarction, arrhythmia, and heart failure reported as serious adverse events; no formal MACE definition.	MI: 1. Cerebral infarction: 1. Chronic heart failure with death: 1. Arrhythmia: 1.	CV events were infrequent and did not allow robust comparisons.
Blauvelt et al., 2017 (VOYAGE 1)^13^	Multinational; 101 centers	Phase III, randomized, double-blind, double-dummy, placebo-controlled with active comparator	Adults with moderate-to-severe plaque psoriasis; IGA ≥ 3, PASI ≥ 12, BSA ≥ 10%; candidates for systemic therapy or phototherapy.	Serious or uncontrolled medical disease, recent malignancy, active TB, previous guselkumab or adalimumab use, or other recent biologics.	837 (334 adalimumab / 174 placebo / 329 guselkumab)	Adalimumab SC: 80 mg week 0; 40 mg week 1; then 40 mg every 2 weeks.	Placebo and guselkumab	48 weeks (16 placebo-controlled)	MACE defined as MI, stroke, or CV death; identified as serious adverse events.	Weeks 0‒16: 1 MI with guselkumab and 1 MI with adalimumab. No additional events through week 48.	MACE rates were low and comparable across groups.
Bachelez et al., 2015 (OPT Compare)^14^	Multinational; 122 centers	Phase III, randomized, double-blind, double-dummy, placebo-controlled	Adults with chronic stable plaque psoriasis; PASI ≥ 12, moderate or severe PGA, BSA ≥ 10%; candidates for systemic therapy or phototherapy.	Non-plaque forms, inability to discontinue previous therapies, previous TNF-i use or non-response, active or clinically significant recent infection.	1106 (336 etanercept / 108 placebo / 659 tofacitinib)	Etanercept SC: 50 mg twice weekly.	Placebo and tofacitinib	12 weeks	MACE defined as MI, stroke/TIA, or CV death; adjudicated by an independent CV committee.	1 MI with tofacitinib 5 mg BID and 1 stroke with etanercept; no CV deaths.	MACE were infrequent and did not allow comparative analyses.
Leonardi et al., 2011 (REACH)^15^	United States and Canada; multicenter outpatient setting	Randomized, double-blind, placebo-controlled, with open-label extension	Adults with moderate-to-severe plaque psoriasis of the hands and/or feet; hfPGA ≥ 3 and additional involvement outside hands/feet.	Previous treatment with adalimumab or palmoplantar pustulosis.	72 (49/23)	Adalimumab SC: 80 mg week 0; then 40 mg every 2 weeks.	Placebo	28 weeks (16 placebo-controlled)	CV events reported as serious adverse events; no formal MACE definition.	1 acute MI with pulmonary edema during the open-label extension with adalimumab. No stroke or CV death.	An isolated event was observed during the open-label extension.
Asahina et al., 2010^16^	Japan; 42 centers	Phase II/III, randomized, double-blind, placebo-controlled	Patients ≥ 20 years with moderate-to-severe plaque psoriasis; PASI ≥ 12 and BSA ≥ 10%; stable disease for ≥ 2 months.	Previous anti-TNF therapy, active infections, relevant autoimmune diseases, cancer, TB, immunosuppression, or hematologic, hepatic, or renal abnormalities.	169 (123 / 46)	Adalimumab SC in three regimens: 40 mg every 2 weeks; 40 mg every 2 weeks with 80 mg loading dose; 80 mg every 2 weeks.	Placebo	24 weeks	CV safety reported as adverse events; no formal MACE definition.	No MI, stroke, AF, heart failure, or thromboembolic events were reported.	No CV events were identified during the double-blind period.
Menter et al., 2008^17^	United States and Canada; 81 centers	Phase III, multicenter, randomized, double-blind, placebo-controlled	Adults with stable, moderate-to-severe plaque psoriasis; BSA ≥ 10%, PASI ≥ 12, and PGA at least moderate.	Symptoms suggestive of demyelinating disease and history of cancer or lymphoproliferative disease, except for some treated localized neoplasms.	1212 (814 / 398)	Adalimumab SC: 80 mg week 0; then 40 mg every 2 weeks.	Placebo	52 weeks (16 placebo-controlled)	Serious CV events, especially heart failure and MI; MACE not prespecified.	1 case of heart failure with adalimumab; no MI or stroke reported.	CV events were limited to one isolated case of heart failure.
Tyring et al., 2007^18^	United States and Canada; 39 centers	Phase III, randomized, double-blind, placebo-controlled, with open-label extension	Adults with active but stable plaque psoriasis, BSA ≥ 10%, PASI ≥ 10, with previous therapy or candidates for systemic therapy or phototherapy.	The report does not fully detail exclusion criteria; concomitant systemic therapy and phototherapy were not allowed.	618 (311 / 307)	Etanercept SC: 50 mg twice weekly.	Placebo	96 weeks (12 placebo-controlled)	Serious CV events such as MI, worsening heart failure, and CV death; no formal MACE definition.	MI: rate 0.4 per 100 patient-years. Two CV deaths during the open-label extension. Worsening heart failure in 2 patients.	CV events were mainly observed during the open-label extension and were infrequent.
Reich et al., 2005 (EXPRESS)^19^	Multinational; 32 centers	Phase III, multicenter, randomized, double-blind, placebo-controlled	Adults with moderate-to-severe plaque psoriasis, PASI ≥ 12, BSA ≥ 10%, candidates for phototherapy or systemic therapy.	Risk of serious infection, lymphoproliferative disease, active TB, or prior treatment with infliximab or other TNF antagonists.	378 (301 / 77)	Infliximab IV: 5 mg/kg at weeks 0, 2, and 6; then every 8 weeks.	Placebo	50 weeks (24 placebo-controlled)	CV events reported as serious adverse events; no formal MACE definition.	No MI, stroke, or de novo heart failure were reported. One non-CV death due to sepsis.	No relevant CV events were identified during follow-up.
Gottlieb et al., 2003^20^	United States; academic and private dermatology centers	Phase II, randomized, double-blind, placebo-controlled	Adults with active stable plaque psoriasis, BSA ≥ 10%, and previous systemic therapy or phototherapy.	Guttate, erythrodermic, or pustular psoriasis, other skin diseases, and medical conditions that could interfere with evaluation.	112 (57 / 55)	Etanercept SC: 25 mg twice weekly.	Placebo	24 weeks	MI and stroke reported among serious adverse events; no formal MACE definition.	1 MI before exposure, excluded from analysis; 1 stroke in placebo during the study; no CV deaths.	No CV events attributable to anti-TNF therapy were observed during treatment.

Part B. Observational studies.											
Study	Country/source	Design	Population	Exclusion criteria	Total N	Anti-TNF	Comparator	Follow-up	CV outcomes	CV results	Main finding
Puig et al 2022^21^	United Kingdom and Ireland; BADBIR	Prospective registry cohort	Patients with psoriasis in BADBIR. Safety set: initiation or switch to SDZ-ADL in the previous 6 months. Comparator cohort: conventional systemics.	Failure to meet operational cohort definitions or absence of PASI/DLQI data for effectiveness.	6055 (136 / 5919)	SDZ-ADL (adalimumab biosimilar); dose according to routine clinical practice.	Conventional systemics	Safety follow-up according to registry risk window; effectiveness median ∼2.0 years.	Serious cardiac events, including MI, stroke, and heart failure; captured through registry reports, hospitalization, and national records.	MI: adjusted HR = 1.71 (95% CI 0.23‒12.53). Stroke: 0 events with SDZ-ADL. Low rates in both groups.	No significant increase in CV risk was observed with SDZ-ADL versus conventional systemics.
Rungapiromnan et al., 2020 (BADBIR)^22^	United Kingdom and Ireland; BADBIR	Prospective comparative new-user cohort	Adults with chronic plaque psoriasis, biologic-naive, enrolled in BADBIR and with at least 6 months of follow-up.	Previous major CV events, age < 18 years, absence of follow-up, prior biologic exposure, or non-eligible treatments.	5468 (4517) TNFi / 951 ustekinumab (secondary analyses with MTX: 2189)	Adalimumab and etanercept in routine clinical practice.	Ustekinumab; secondary comparisons with adalimumab and methotrexate	Median ∼2 years (up to 3.2 years depending on drug)	MACE defined as acute coronary syndrome, unstable angina, MI, or stroke; clinically validated.	Adjusted HR ustekinumab vs. TNFi: 0.96 (95% CI 0.41‒2.22). Secondary comparisons were also non-significant.	No significant differences in MACE risk were identified after adjustment for confounders.
Shaaban et al., 2018^23^	Kuwait; hospital EHR	Retrospective cohort	Adults with psoriasis treated with TNF-i, methotrexate, or topical therapy only; exposures of at least 3 months for TNF-i and methotrexate.	Documented MI before January 1, 2008.	4762 (1058 TNFi / 1331 MTX / 2373 topicals)	Adalimumab, etanercept, or infliximab for at least 3 months.	Methotrexate or topical treatment only	Median 3.9 years	Acute MI requiring hospitalization; identified in electronic health records.	MI: 1.79% with TNFi, 3.03% with MTX, and 3.03% with topicals. TNFi vs. topicals: p = 0.01. TNFi vs. MTX: non-significant.	Anti-TNF therapy was associated with a lower absolute frequency of MI versus topicals, without a clear difference versus MTX.
Wu et al., 2012^24^	United States; Kaiser Permanente Southern California	Population-based retrospective cohort	Patients with ≥ 3 codes for psoriasis or psoriatic arthritis, membership ≥ 1 year, and at least one annual medical encounter; TNFi, systemic/phototherapy, or topical cohorts.	Previous MI before the third diagnostic code for psoriasis or psoriatic arthritis.	8845 (1673 TNFi / 2097 systemic-phototherapy / 5075 topicals)	Etanercept, infliximab, or adalimumab for at least 2 months.	Oral systemics/phototherapy or topical treatment	Median 4.3 years (IQR 2.9‒5.5)	Incident fatal or non-fatal MI; identified by ICD-9 codes and death certificates.	TNFi vs. topicals: adjusted HR 0.50 (95% CI 0.32‒0.79). TNFi vs. systemic/phototherapy: non-significant.	Anti-TNF therapy was associated with a lower risk of MI versus topicals, but not versus systemic therapy/phototherapy.

AF, Atrial Fibrillation; BADBIR, British Association of Dermatologists Biologic and Immunomodulators Register; BSA, Body Surface Area; CV, Cardiovascular; DLQI, Dermatology Life Quality Index; HR, Hazard Ratio; ICD-9, International Classification of Diseases, Ninth Revision; IGA, Investigator Global Assessment; IQR, Interquartile Range; MACE, Major Adverse Cardiovascular Events; MI, Myocardial Infarction; mNAPSI, modified Nail Psoriasis Severity Index; MTX, Methotrexate; PASI, Psoriasis Area and Severity Index; PGA, Physician Global Assessment; SC, Subcutaneous; TIA, Transient Ischemic Attack; TNF-i, Tumor Necrosis Factor inhibitor.

In this review, the primary cardiovascular outcomes of interest were MACE, myocardial infarction, stroke, and cardiovascular death. MACE was explicitly evaluated in only a limited proportion of studies,^13^, ^14^, ^22^ using composite definitions based on myocardial infarction, stroke, and/or cardiovascular death in both clinical trials^13^, ^14^ and one prospective cohort.^22^ In these studies, events were infrequent and no relevant between-group differences were observed.^13^, ^14^, ^22^ Myocardial infarction was the most frequently reported individual outcome, although it remained rare in the clinical trials.^12^, ^15^, ^18^ In observational studies, findings were heterogeneous: Wu et al.^24^ reported a lower rate of myocardial infarction with TNF inhibitors compared with topical therapy, Shaaban et al.^23^ reported a lower frequency of this outcome with TNF inhibitors than with methotrexate and topical treatments, whereas Puig et al.^21^ did not find a significant increase in risk versus conventional systemic therapies. Stroke was mainly reported as an isolated event^12^, ^14^, ^20^ and was not consistently increased in observational studies.^21^, ^22^ Cardiovascular death was exceptionally rare, with no cases reported by Blauvelt et al.,^13^ Bachelez et al.,^14^ Menter et al.,^17^ or Gottlieb et al.,^20^ and only two events described during the open-label extension of Tyring et al.^18^ Cai et al.^12^ additionally reported one case of chronic heart failure with a fatal outcome. Regarding secondary outcomes, all-cause mortality was infrequent and was mainly reported as isolated events, including one non-cardiovascular death due to sepsis^19^ and one death associated with chronic heart failure.^12^ Heart failure was reported as a serious adverse event, with isolated cases or clinical worsening in some studies,^11^, ^12^, ^15^, ^17^^,^^18^ whereas other studies reported no cases.^16^, ^19^ Atrial fibrillation was documented in only one case, which occurred in the placebo group in the study by Elewski et al.^11^ Venous thromboembolism was the least frequently documented outcome, with only one study explicitly reporting the absence of thromboembolic events.^16^

#### Risk of bias assessment

Among the randomized clinical trials, most studies were judged to have an overall risk of bias of “some concerns” according to RoB 2.^11^, ^12^, ^13^, ^14^, ^16^, ^17^, ^18^, ^19^, ^20^ In general, this was driven by concerns in domains related to deviations from intended interventions, missing outcome data, outcome measurement, or selective reporting, whereas the randomization process showed a low risk of bias in most studies.^11^, ^12^, ^13^, ^14^, ^16^, ^17^, ^18^, ^19^, ^20^ The main exception was the study by Leonardi et al.,^15^ which was judged to have a high overall risk of bias, mainly because of limitations in the selective reporting domain. In the observational studies,^21^, ^22^, ^23^, ^24^ the risk of bias was generally higher than in the clinical trials.^11^, ^12^, ^13^, ^14^, ^16^, ^17^, ^18^, ^19^, ^20^ The studies by Puig et al.^21^ and Rungapiromnan et al.^22^ were rated as having a moderate overall risk of bias, whereas the studies by Shaaban et al.^23^ and Wu et al.^24^ were judged to have a serious overall risk of bias according to ROBINS-I (Table 2, Table 3).

**Table 2:** Risk of bias assessment of randomized clinical trials using RoB2.^11^, ^12^, ^13^, ^14^, ^15^, ^16^, ^17^, ^18^, ^19^, ^20^

Study and year	Design / Source	Exposure vs. comparator	Outcome	D1: Randomization process	D2: Deviations from intended interventions	D3: Missing outcome data	D4: Measurement of the outcome	D5: Selection of the reported result	Overall judgment
Elewski et al. 2018^11^	Double-blind RCT; escape to open-label phase	Adalimumab vs. placebo	Serious cardiovascular events (safety)	Low	Some concerns	Some concerns	Some concerns	Some concerns	Some concerns
Blauvelt et al. 2017 (VOYAGE 1)^13^	Phase 3 RCT; double-blind; crossover from placebo	Adalimumab vs. comparators	MACE (defined) / rare events	Low	Low	Some concerns	Some concerns	Some concerns	Some concerns
Cai et al. 2017^12^	Phase 3 RCT; 12-week double-blind + 12-week open-label	Adalimumab vs. placebo; thereafter all ADA	Serious cardiovascular events (including 1 death due to heart failure)	Low	Some concerns	Some concerns	Some concerns	Some concerns	Some concerns
Bachelez et al. 2015 (OPT Compare)^14^	Double-dummy RCT	Etanercept vs. placebo	MACE (defined and adjudicated)	Low	Low	Low	Low	Some concerns	Some concerns
Leonardi et al. 2011 (REACH)^15^	RCT; 16-week double-blind + 12-week open-label; exclusion of 1 center (n = 9)	Adalimumab vs. placebo; thereafter all ADA	Serious cardiovascular events (extension)	Low	Some concerns	Some concerns	Some concerns	High	High
Asahina et al. 2010^16^	Double-blind RCT	Adalimumab vs. placebo	PASI (primary); no MACE	Low	Some concerns	Some concerns	Low	Some concerns	Some concerns
Menter et al. 2008^17^	52-week RCT; DBPC 0–16 + open-label phase + rerandomization	Adalimumab vs. placebo and rerandomization	Heart failure as an adverse event of interest	Low	Some concerns	Some concerns	Some concerns	Some concerns	Some concerns
Tyring et al. 2007^18^	RCT DBPC 0–12 + open-label extension to 96 weeks	Etanercept vs. placebo; thereafter all ETN	Serious cardiovascular events (MI, probable cardiovascular deaths, worsening heart failure)	Low	Some concerns	Some concerns	Some concerns	Some concerns	Some concerns
Reich et al. 2005 (EXPRESS)^19^	Phase 3 RCT; DBPC 0–24 with crossover	Infliximab vs. placebo; thereafter placebo to IFX	Heart failure / safety (no formal MACE)	Low	Some concerns	Some concerns	Some concerns	Some concerns	Some concerns
Gottlieb et al. 2003^20^	24-week double-blind RCT	Etanercept vs. placebo	Serious cardiovascular events reported as serious adverse events	Low	Low	Some concerns	Some concerns	Some concerns	Some concerns

ADA, Adalimumab; DBPC, Double-Blind Placebo-Controlled; ETN, Etanercept; IFX, Infliximab; MACE, Major Adverse Cardiovascular events; MI, Myocardial Infarction; PASI, Psoriasis Area and Severity Index; RCT, Randomized Controlled Trial; REACH, Randomized Evaluation of Adalimumab in Chronic Plaque Psoriasis of the Hands and Feet; RoB2, Risk of Bias 2; VOYAGE 1, phase III study of guselkumab versus adalimumab and placebo in moderate-to-severe psoriasis.

**Table 3:** Risk of bias assessment of observational studies using ROBINS-I.^21^, ^22^, ^23^, ^24^

Study and year	Design / source	Exposure vs comparator	Outcome	D1: Confounding	D2: Selection of participants	D3: Classification of interventions	D4: Deviations from intended interventions	D5: Missing data	D6: Measurement of outcomes	D7: Selection of the reported result	Overall judgment
Puig et al. 2022^21^	Prospective BADBIR registry (UK/ROI)	SDZ-ADL vs. conventional systemics	MI / serious cardiac adverse event	Moderate	Moderate	Low	Low	Low	Moderate	Moderate	Moderate
Rungapiromnan et al. 2020^22^	Prospective cohort (BADBIR registry; 2007–2016)	Ustekinumab vs. TNFi (including comparisons with adalimumab and MTX)	Major cardiovascular events (ACS/ UA/ MI/ Stroke)	Moderate	Moderate	Low	Low	Low	Low	Moderate	Moderate
Shaaban et al. 2018^23^	Retrospective cohort (Farwaniya Hospital EHR, Kuwait; 2008–2014)	TNFi ≥3 months vs. MTX ≥ 3 months vs. topical therapies	Hospitalized myocardial infarction	Serious	Moderate	Moderate	Moderate	Moderate	Moderate	Moderate	Serious
Wu et al. 2012^24^	Retrospective cohort (KPSC; 2004–2010)	TNFi ≥2 months vs. topical / oral treatment-phototherapy	Incident myocardial infarction	Serious	Moderate	Moderate	Moderate	Moderate	Moderate	Moderate	Serious

ACS, Acute Coronary Syndrome; BADBIR, British Association of Dermatologists Biologic and Immunomodulators Register; EHR, Electronic Health Record; KPSC, Kaiser Permanente Southern California; MI, Myocardial Infarction; MTX, Methotrexate; ROBINS-I, Risk Of Bias In Non-randomized Studies of Interventions; ROI, Republic of Ireland; SDZ-ADL, Adalimumab biosimilar; TNFi, Tumor Necrosis Factor Inhibitors; UA, Unstable Angina; UK, United Kingdom.

### Quantitative review

#### Primary outcomes

##### MACE

In clinical trials, MACE did not differ significantly between groups over a follow-up period of 12- to 24-weeks, with 6 events among 1,112 patients treated with anti-TNF therapy versus 1 event among 446 patients in the placebo group (RR = 1.11; 95% CI 0.30–4.15; *I*^2^ = 0%) (Fig. 2), with very low certainty of evidence (Table 4).^12^, ^13^, ^14^, ^15^, ^20^ Sensitivity analysis showed consistent results when studies of adalimumab (RR = 1.73; 95% CI 0.30–10.07)^12^, ^13^, ^15^ and etanercept (RR = 0.53; 95% CI 0.06–4.72)^14^, ^20^ were analyzed separately.

**Figure 2:**
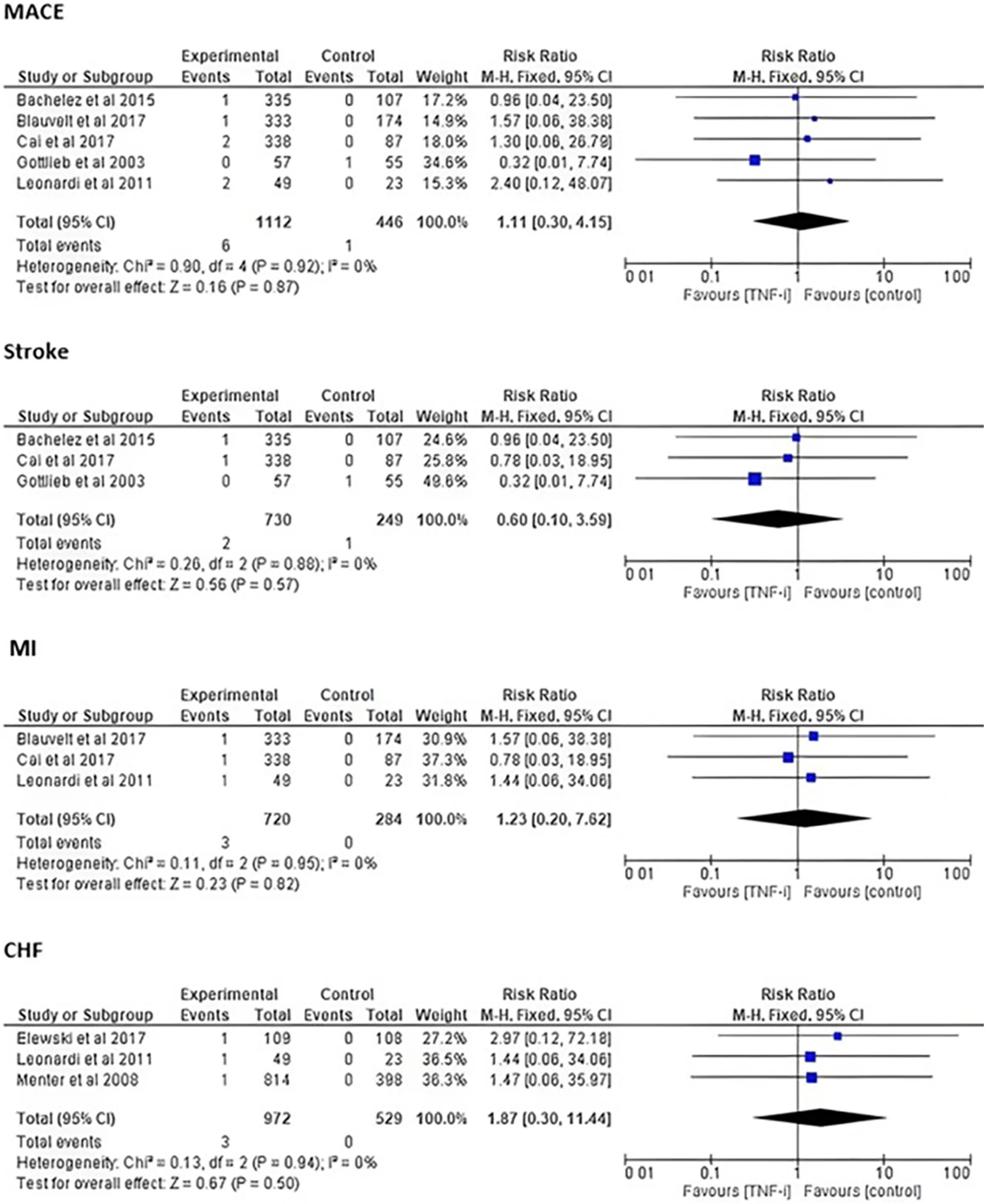
Forest plots of Major Adverse Cardiovascular Events (MACE), stroke, Myocardial Infarction (MI), and Congestive Heart Failure (CHF) in clinical trials of TNF inhibitors versus control.

**Table 4:** GRADE certainty of evidence for cardiovascular outcomes associated with TNF inhibitors in adults with plaque psoriasis.

Certainty assessment							No. of patients		Effect		Certainty	Importance
No. of studies	Study design	Risk of bias	Inconsistency	Indirectness	Imprecision	Other considerations	Intervention	Comparison	Relative effect (95% CI)	Absolute effect (95% CI)		
**MACE**												
5	Randomized trials	Seriousᵃ	Not serious	Serious^b^	Serious^c^	None	6/1112 (0.5%)	1/446 (0.2%)	RR 1.11 (0.30 to 4.15)	0 fewer per 1,000 (from 2 fewer to 7 more)	⨁◯◯◯ Very low ^a,b,c^	CRITICAL
**MI**												
3	Randomized trials	Seriousᵃ	Not serious	Serious^b^	Serious^c^	None	3/720 (0.4%)	0/284 (0.0%)	RR 1.23 (0.20 to 7.62)	0 fewer per 1,000 (from 0 fewer to 0 fewer)	⨁◯◯◯ Very low ^a,b,c^	CRITICAL
**Stroke**												
3	Randomized trials	Serious^d^	Not serious	Serious^b^	Serious^c^	None	2/730 (0.3%)	1/249 (0.4%)	RR 0.60 (0.10 to 3.59)	2 fewer per 1,000 (from 4 fewer to 10 more)	⨁◯◯◯ Very low ^b,c,d^	CRITICAL
**CHF**												
3	Randomized trials	Seriousᵃ	Not serious	Serious^b^	Serious^c^	None	3/972 (0.3%)	0/529 (0.0%)	RR 1.87 (0.30 to 11.44)	0 fewer per 1,000 (from 0 fewer to 0 fewer)	⨁◯◯◯ Very low ^a,b,c^	IMPORTANT
**MACE – TNFi vs topical therapy – long-term observational studies**												
2	Observational studies	Very serious^e^	Not serious	Not serious	Not serious	None	47/2731 (1.7%)	224/7448 (3.0%)	RR 0.57 (0.42 to 0.78)	13 fewer per 1,000 (from 17 fewer to 7 fewer)	⊕⊕◯◯ Low^e^	CRITICAL
**MACE – TNFi vs oral treatment/phototherapy – long-term observational studies**												
4	Observational studies	Very serious^e^	Not serious	Not serious	Not serious	None	71/6071 (1.2%)	165/11536 (1.4%)	RR 1.04 (0.75 to 1.45)	1 more per 1,000 (from 3 fewer to 7 more)	⊕⊕◯◯ Low^e^	CRITICAL

CI, Confidence Interval; RR, Risk Ratio.

Explanations:

a Downgraded for risk of bias due to one trial at high risk and several trials with some concerns.

^b^ Downgraded for indirectness because cardiovascular outcomes were assessed mainly as safety events, not as primary endpoints.

^c^ Downgraded for imprecision due to few events, limited statistical power, and wide confidence intervals.

^d^ Downgraded for risk of bias because the overall RoB 2 judgment was some concerns.

^e^ Downgraded two levels for risk of bias in observational studies, mainly due to residual confounding and confounding by indication.

In observational studies, when stratified by comparator, the pooled risk of reported cardiovascular events was lower in patients treated with anti-TNF therapy than in those receiving topical treatment, with 47 events among 2,731 patients versus 224 events among 7,448 patients (RR = 0.57; 95% CI 0.42–0.78; *I*^2^ = 0%) (Fig. 3), with low certainty of evidence (Table 4).^23^, ^24^ Sensitivity analyses performed by sequential exclusion of each study confirmed the consistency of this association, with RR estimates of 0.56 (95% CI 0.37–0.83)^24^ and 0.59 (95% CI 0.36–0.98).^23^

**Figure 3:**
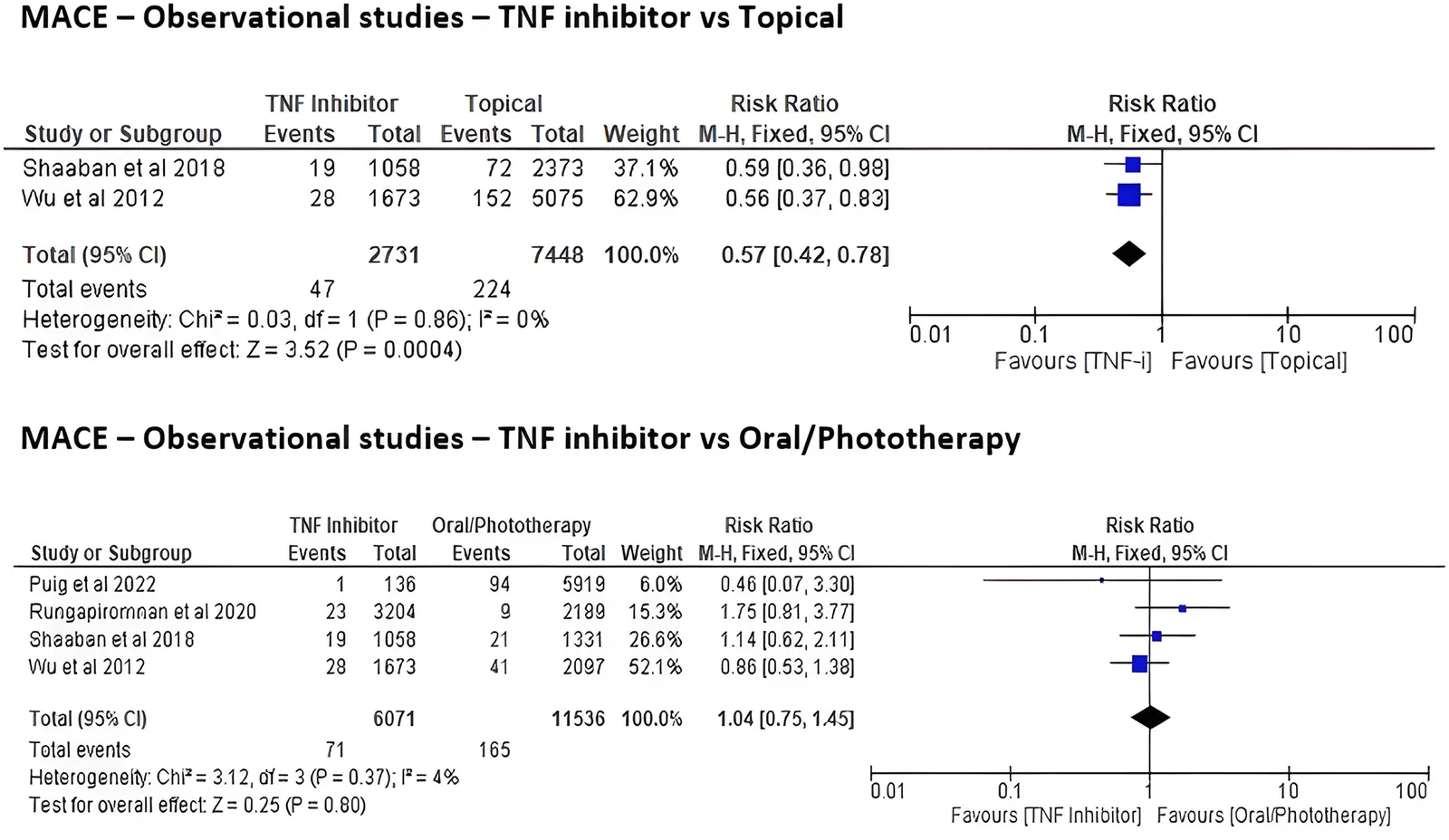
Forest plots of pooled cardiovascular event analyses in observational studies comparing TNF inhibitors with topical therapy and with oral therapy/phototherapy.

In contrast, when anti-TNF therapy was compared with oral treatment or phototherapy in studies with a median follow-up of 2.0- to 4.3-years, no significant differences were observed, with 71 events among 6,071 patients versus 165 events among 11,536 patients (RR = 1.04; 95% CI 0.75–1.45; *I*^2^ = 4%) (Fig. 3), with low certainty of evidence (Table 4).^21^, ^22^, ^23^, ^24^ Sensitivity analyses, also performed by sequential exclusion of each study, yielded consistent results and did not meaningfully modify the effect estimate, with RR values of 1.08 (95% CI 0.77–1.51),^22^, ^23^, ^24^ 0.92 (95% CI 0.63–1.33),^21^, ^23^, ^24^ 1.01 (95% CI 0.68–1.49),^21^, ^22^, ^24^ and 1.25 (95% CI 0.79–1.98).^21^, ^22^, ^23^

##### Myocardial infarction

In clinical trials, all data for the outcome of myocardial infarction came from studies comparing adalimumab with placebo. During a follow-up period of 12- to 16-weeks, no significant differences were observed between groups, with 3 events among 720 patients treated with adalimumab and no events among 284 patients in the placebo group (RR = 1.23; 95% CI 0.20–7.62; *I*^2^ = 0%) (Fig. 2), with very low certainty of evidence (Table 4).^12^, ^13^, ^15^ Sensitivity analyses performed by sequential exclusion of each study confirmed the absence of a significant association, with RR estimates of 1.08 (95% CI 0.12–10.02),^12^, ^15^ 1.50 (95% CI 0.16–14.25),^13^, ^15^ and 1.14 (95% CI 0.12–10.60).^12^, ^13^

##### Stroke

In clinical trials, the outcome of stroke did not differ significantly between groups during a follow-up period of 12- to 24-weeks. The analysis included studies comparing adalimumab or etanercept with placebo and recorded 2 events among 730 patients in the anti-TNF group versus 1 event among 249 patients in the placebo group (RR = 0.60; 95% CI 0.10–3.59; *I*^2^ = 0%) (Fig. 2), with very low certainty of evidence (Table 4).^12^, ^14^, ^20^ In the sensitivity analysis, the absence of statistically significant differences remained when the studies evaluating etanercept alone (RR = 0.53; 95% CI 0.06–4.72)^14^, ^20^ and the study evaluating adalimumab (RR = 0.78; 95%CI 0.03–18.95)^12^ were analyzed separately.

##### Cardiovascular death

Insufficient data were available.

#### Secondary outcomes

##### Heart failure

In clinical trials, the outcome of congestive heart failure did not differ significantly between groups during a follow-up period of 16- to 26-weeks. All included studies compared adalimumab with placebo and recorded 3 events among 972 patients in the adalimumab group versus no events among 529 patients in the placebo group (RR = 1.87; 95% CI 0.30–11.44; *I*^2^ = 0%) (Fig. 2), with very low certainty of evidence (Table 4).^11^, ^15^, ^17^ The result was consistent in the sensitivity analysis, with RR estimates of 1.45 (95% CI 0.15–13.79),^15^, ^17^ 2.11 (95% CI 0.23–19.54),^11^, ^17^ and 2.09 (95% CI 0.23–19.11).^11^, ^15^

##### All-cause mortality, atrial fibrillation, and venous thromboembolism

Insufficient data were available.

## Discussion

The findings of this review suggest that anti-TNF agents were not associated with a clear increase in the risk of major cardiovascular events in patients with moderate-to-severe plaque psoriasis, supporting an overall acceptable short-term cardiovascular safety profile. This is clinically relevant because anti-TNF agents are used in a population characterized by persistent systemic inflammation and an increased cardiovascular risk burden. However, the absence of statistically significant differences should be interpreted cautiously, as the low frequency of events and short follow-up in the included trials reduce both the precision of the estimates and the ability to detect modest but clinically meaningful effects on hard cardiovascular outcomes.^12^, ^13^, ^14^, ^15^, ^20^ In observational analyses stratified by comparator, the pooled risk was lower with anti-TNF therapy than with topical treatment,^23^, ^24^ but not when compared with oral treatment or phototherapy.^21^, ^22^, ^23^, ^24^ This pattern probably reflects differences in comparator choice, background disease severity, and residual confounding rather than a true biological inconsistency. In particular, confounding by indication, baseline differences in psoriasis severity, and variation in cardiovascular risk management may have influenced the observational estimates.^23^, ^24^ Overall, the findings of the present review support relative cardiovascular safety rather than conclusive cardioprotection.

These results are broadly consistent with previous systematic reviews and meta-analyses. Mangkorntongsakul et al. found no significant differences in the risk of MACE between biologics and placebo, nor in the anti-TNF subgroup, which is consistent with the findings of this review for randomized evidence.^25^ Similarly, Rungapiromnan et al. reported no significant differences in the risk of MACE for biologics overall or for anti-TNF agents.^26^ Taken together, this consistency reinforces the absence of a clear signal of major cardiovascular harm with anti-TNF therapy in the short term. Nevertheless, the present review offers a more specific interpretation by showing that the favorable signal observed in real-world studies was not uniform across all comparators, suggesting that any potential cardiovascular benefit should be interpreted within the therapeutic and methodological context of each study.

The recent systematic review and meta-analysis by Galajda et al. evaluated TNF inhibitors across immune-mediated inflammatory diseases and reported a reduced risk of MACE compared with conventional systemic non-biologic therapies overall and in the psoriasis/psoriatic arthritis subgroup.^7^ The present findings provide a more disease-specific and outcome-specific synthesis focused on adults with moderate-to-severe plaque psoriasis. Several methodological differences may explain the more conservative interpretation of the present review. Galajda et al. pooled adjusted hazard ratios and incidence rate ratios from largely observational evidence across broader immune-mediated inflammatory disease populations, whereas this review separated randomized and observational evidence, restricted eligibility to plaque psoriasis, included placebo-controlled trial data, stratified observational analyses by comparator type, and prioritized clinical cardiovascular outcomes defined or reported within psoriasis-specific cohorts.

This review has several limitations. First, most of the available evidence was derived from clinical trials that were not specifically designed to evaluate cardiovascular outcomes, but rather dermatologic efficacy and general safety, which reduces the power to detect differences in infrequent events such as MACE, myocardial infarction, or stroke.^12^, ^13^, ^14^, ^15^, ^20^ In addition, follow-up duration was generally short, meaning that the randomized evidence is more informative for short-term cardiovascular safety than for the potential effect of anti-TNF agents on late atherothrombotic outcomes.^12^, ^13^, ^15^, ^20^ Several analyses were also based on a limited number of studies and events, which decreases the precision of the estimates and requires cautious interpretation of the findings.^12^, ^13^, ^14^, ^15^ Another important limitation is the heterogeneity in the definition and reporting of cardiovascular outcomes across the included studies. In some studies, MACE was evaluated as an explicit composite outcome, whereas in others cardiovascular events were reported as serious adverse events or as isolated outcomes, thereby limiting comparability across studies and the consistency of the pooled syntheses. In addition, the observational evidence was subject to residual confounding and confounding by indication, which restrict the ability to establish firm causal relationships between anti-TNF exposure and the observed cardiovascular outcomes.^21^, ^22^, ^23^, ^24^

From a clinical perspective, these findings suggest that anti-TNF agents may be considered a reasonable option in patients with moderate-to-severe psoriasis, without consistent evidence of short-term cardiovascular harm. However, treatment selection should not be based on an expectation of proven cardioprotection, but rather on a comprehensive assessment of efficacy, comorbidities, and each patient’s overall safety profile.^5^ Furthermore, the management of these patients should include systematic evaluation and control of cardiovascular risk factors, since overall risk reduction likely depends both on the control of systemic inflammation and on appropriate management of cardiometabolic comorbidities.^27^

## Conclusion

Anti-TNF agents were not associated with a clear increase in the risk of major cardiovascular events in patients with moderate-to-severe plaque psoriasis. Current evidence supports an acceptable short-term cardiovascular safety profile, although it does not establish a conclusive cardioprotective effect. Larger studies with longer follow-up are needed to define their impact on clinical cardiovascular outcomes more precisely.

## ORCID IDs

Luisa Fernanda Mesa Rodríguez: 0009-0002-2956-7468

Alejandro Zambrano Aristizábal: 0009-0006-9436-4201

## Research data availability

The entire dataset supporting the results of this study was published in this article.

## Financial support

None declared.

## Authors’ contributions

Luisa Fernanda Mesa Rodríguez: Study concept and design; data collection, analysis and interpretation of data; statistical analysis; writing of the manuscript and critical review of important intellectual content; effective participation in the research guidance; critical review of the literature; final approval of the final version of the manuscript.

Alejandro Zambrano Aristizábal: Study concept and design; data collection, analysis and interpretation of data; statistical analysis; writing of the manuscript and critical review of important intellectual content; effective participation in the research guidance; critical review of the literature; final approval of the final version of the manuscript.

Juan David Vega Padilla: Study concept and design; data collection, analysis and interpretation of data; statistical analysis; writing of the manuscript and critical review of important intellectual content; effective participation in the research guidance; critical review of the literature; final approval of the final version of the manuscript.

## Conflicts of interest

None declared.
