# Supplementary material for: Tumor necrosis factor inhibitors and cardiovascular outcomes in adults with moderate-to-severe plaque psoriasis: a systematic review and meta-analysis
Source: An Bras Dermatol. 2026 Oct 27;101(6):501473. doi: 10.1016/j.abd.2026.501473 (PMC13735168; doi:10.1016/j.abd.2026.501473)
Supplement: Supplementary file 1 [file mmc1.docx]

**ABD-D-26-00329_Supplementary Material**

**Supplementary Table 1 Excluded studies and reasons for exclusion.**

This supplementary table lists the studies excluded after full-text review or after assessment from other sources.

| **Supplementary ref.** | **Study** | **Reason for exclusion** |
| --- | --- | --- |
| 1 | Elnabawi et al. 2019 | Did not report clinical cardiovascular events; it evaluated coronary plaque characteristics, corresponding to an imaging surrogate outcome. |
| 2 | Elnabawi et al. 2019 | The primary outcome was coronary inflammation measured by the perivascular fat attenuation index on CCTA, corresponding to an imaging biomarker rather than a clinical event. |
| 3 | Blauvelt et al. 2018 | Did not address the PICO question, as it did not compare cardiovascular outcomes between anti-TNF and non-anti-TNF therapies. |
| 4 | Wu et al. 2018 | The population was not eligible, as it included patients with psoriasis and psoriatic arthritis without psoriasis-specific effect estimates for moderate-to-severe plaque psoriasis. |
| 5 | Caiazzo et al. 2018 | Narrative review; not an original study. |
| 6 | Lebwohl 2017 | Editorial commentary; not an original study. |
| 7 | Richter et al. 2017 | Did not include a non-anti-TNF or non-biologic comparator. |
| 8 | Bissonnette et al. 2017 | Full-text access could not be obtained. |
| 9 | Kim et al. 2016 | Retrospective cohort including only patients exposed to biologics; it did not include a non-anti-TNF control group or formal comparators with conventional systemic or topical/UV therapies. |
| 10 | Gulliver et al. 2016 | Grouped anti-IL-12/23 and anti-TNF therapies within the same analysis, precluding a specific comparison for anti-TNF agents. |
| 11 | Strober et al. 2016 | Did not evaluate MACE, myocardial infarction, stroke, or cardiovascular death as outcomes of interest. |
| 12 | Papp et al. 2016 | Post hoc analysis of three clinical trials, two of which had already been excluded individually. |
| 13 | Kimball et al. 2015 | Did not include an internal non-anti-TNF comparator. |
| 14 | Pina et al. 2015 | Six-month prospective single-arm study that evaluated metabolic and inflammatory surrogate outcomes, without a non-anti-TNF comparator group or reporting incident clinical cardiovascular events with effect estimates. |
| 15 | Bissonnette et al. 2013 | The primary outcome was a surrogate measure (arterial inflammation assessed by 18F-FDG PET/CT); it did not evaluate MACE. |
| 16 | Leonardi et al. 2012 | Did not include a comparator, as all patients received adalimumab within a treatment program. |
| 17 | Papp et al. 2012 | Post hoc analysis of patients treated with etanercept from phase III trials and open-label extensions, without a non-anti-TNF comparator group in the analysis. |
| 18 | Papp et al. 2011 | Review article summarizing trials of etanercept; not an original study. |
| 19 | Ortonne et al. 2010 | Ineligible comparator: in BELIEVE, all patients received adalimumab and there was no non-anti-TNF study arm. |
| 20 | Vilar et al. 2010 | Available only as an abstract, without sufficient data for full assessment and extraction. |
| 21 | Papp et al. 2009 | Reported duplicate data. |
| 22 | Zachariae et al. 2008 | Did not provide an eligible anti-TNF versus non-anti-TNF comparator for the review question. |
| 23 | Song et al. 2025 | Anti-TNF agents were included within a general biologics group and no class-specific anti-TNF analysis was available. |
| 24 | Ahn et al. 2025 | Anti-TNF agents were included within a general biologics group and no class-specific anti-TNF analysis was available. |
| 25 | Lluch-Galcerá et al. 2025 | Full-text access was not available. |
| 26 | Pina Vegas et al. 2022 | The comparator included another biologic, making the anti-TNF versus non-anti-TNF comparison ineligible. |
| 27 | Cho et al. 2024 | The comparator included another biologic class, making the anti-TNF versus non-anti-TNF comparison ineligible. |
| 28 | Lee et al. 2019 | The comparator included another biologic, making the anti-TNF versus non-anti-TNF comparison ineligible. |
| 29 | Kristensen et al. 2023 | The anti-TNF versus non-anti-TNF comparison was not evaluated. |
| 30 | Kridin et al. 2025 | The anti-TNF versus non-anti-TNF comparison was not evaluated. |
| 31 | Saurat et al. 2008 | Clinically relevant cardiovascular events were not reported. |
| 32 | Gordon et al. 2006 | The anti-TNF versus non-anti-TNF comparison was not evaluated. |

**Supplementary references**

1. Elnabawi YA, Dey AK, Goyal A, Groenendyk JW, Chung JH, Belur AD, et al. Coronary artery plaque characteristics and treatment with biologic therapy in severe psoriasis: results from a prospective observational study. Cardiovasc Res. 2019;115:721-8.

2. Elnabawi YA, Oikonomou EK, Dey AK, Mancio J, Rodante JA, Aksentijevich M, et al. Association of biologic therapy with coronary inflammation in patients with psoriasis as assessed by perivascular fat attenuation index. JAMA Cardiol. 2019;4:885-91.

3. Blauvelt A, Lacour JP, Fowler JF Jr, Weinberg JM, Gospodinov D, Schuck E, et al. Phase III randomized study of the proposed adalimumab biosimilar GP2017 in psoriasis: impact of multiple switches. Br J Dermatol. 2018;179:623-31.

4. Wu JJ, Joshi AA, Reddy SP, Batech M, Egeberg A, Ahlehoff O, et al. Anti-inflammatory therapy with tumour necrosis factor inhibitors is associated with reduced risk of major adverse cardiovascular events in psoriasis. J Eur Acad Dermatol Venereol. 2018;32:1320-6.

5. Caiazzo G, Fabbrocini G, Di Caprio R, Raimondo A, Scala E, Balato N, et al. Psoriasis, cardiovascular events, and biologics: lights and shadows. Front Immunol. 2018;9:1668.

6. Lebwohl M. Does treatment of psoriasis reduce cardiovascular comorbidities? J Invest Dermatol. 2017;137:1612-3.

7. Richter L, Vujic I, Sesti A, Monshi B, Sanlorenzo M, Posch C, et al. Etanercept, adalimumab, and ustekinumab in psoriasis: analysis of 209 treatment series in Austria. J Dtsch Dermatol Ges. 2017;15:309-17.

8. Bissonnette R, Kerdel F, Naldi L, Papp K, Galindo C, Langholff W, et al. Evaluation of risk of major adverse cardiovascular events with biologic therapy in patients with psoriasis. J Drugs Dermatol. 2017;16:1002-13.

9. Kim WB, Qiang J, Yeung J. MACEs not associated with the use of biologic therapy for psoriasis in real-world clinical practice: a Canadian multicenter retrospective study. J Cutan Med Surg. 2016;20:352-3.

10. Gulliver WP, Randell S, Gulliver S, Connors S, Bachelez H, MacDonald D, et al. Do biologics protect patients with psoriasis from myocardial infarction? A retrospective cohort. J Cutan Med Surg. 2016;20:536-41.

11. Strober BE, Bissonnette R, Fiorentino D, Kimball AB, Naldi L, Shear NH, et al. Comparative effectiveness of biologic agents for the treatment of psoriasis in a real-world setting: results from a large, prospective, observational study (Psoriasis Longitudinal Assessment and Registry [PSOLAR]). J Am Acad Dermatol. 2016;74:851-61.

12. Papp KA, Armstrong AW, Reich K, Karunaratne M, Valdecantos W. Adalimumab efficacy in patients with psoriasis who received or did not respond to prior systemic therapy: a pooled post hoc analysis of results from three double-blind, placebo-controlled clinical trials. Am J Clin Dermatol. 2016;17:79-86.

13. Kimball AB, Rothman KJ, Kricorian G, Pariser D, Yamauchi PS, Menter A, et al. OBSERVE-5: observational postmarketing safety surveillance registry of etanercept for the treatment of psoriasis final 5-year results. J Am Acad Dermatol. 2015;72:115-22.

14. Pina T, Armesto S, Lopez-Mejias R, Genre F, Ubilla B, Gonzalez-Lopez MA, et al. Anti-TNF-α therapy improves insulin sensitivity in non-diabetic patients with psoriasis: a 6-month prospective study. J Eur Acad Dermatol Venereol. 2015;29:1325-30.

15. Bissonnette R, Tardif JC, Harel F, Pressacco J, Bolduc C, Guertin MC. Effects of the tumor necrosis factor-α antagonist adalimumab on arterial inflammation assessed by positron emission tomography in patients with psoriasis: results of a randomized controlled trial. Circ Cardiovasc Imaging. 2013;6:83-90.

16. Leonardi C, Sobell JM, Crowley JJ, Mrowietz U, Bao Y, Mulani PM, et al. Efficacy, safety and medication cost implications of adalimumab 40 mg weekly dosing in patients with psoriasis with suboptimal response to 40 mg every other week dosing: results from an open-label study. Br J Dermatol. 2012;167:658-67.

17. Papp KA, Poulin Y, Bissonnette R, Bourcier M, Toth D, Rosoph L, et al. Assessment of the long-term safety and effectiveness of etanercept for the treatment of psoriasis in an adult population. J Am Acad Dermatol. 2012;66:e33-e45.

18. Papp KA, Krueger GG, Jemec GB, Van De Kerkhof P, Robertson D, Estojak J, et al. Long-term, continuous dosing of etanercept in patients with plaque psoriasis. Expert Rev Dermatol. 2011;6:361-73.

19. Ortonne JP, Chimenti S, Reich K, Gniadecki R, Sprøgel P, Unnebrink K, et al. Efficacy and safety of adalimumab in patients with psoriasis previously treated with anti-tumour necrosis factor agents: subanalysis of BELIEVE. J Eur Acad Dermatol Venereol. 2011;25:1012-20.

20. Vilar J, Dehesa LA, Valerón P, Santana N, Carretero G. Our experiences using adalimumab in a psoriasis unit: P 064. J Eur Acad Dermatol Venereol. 2010;24:29.

21. Papp K, Okun M, Vender R. Adalimumab in the treatment of psoriasis: pooled efficacy and safety results from three pivotal studies. J Cutan Med Surg. 2009;13 Suppl 2:S58-S66.

22. Zachariae C, Mørk NJ, Reunala T, Lorentzen H, Falk E, Karvonen SL, et al. The combination of etanercept and methotrexate increases the effectiveness of treatment in active psoriasis despite inadequate effect of methotrexate therapy. Acta Derm Venereol. 2008;88:495-501.

23. Song WJ, Oh S, Yoon HS. Association between biologic and nonbiologic systemic therapy for psoriasis and psoriatic arthritis and the risk of new-onset and recurrent major adverse cardiovascular events: a retrospective cohort study. J Am Acad Dermatol. 2025;93:141-9.

24. Ahn HK, Oh S, Yoon HS. Treatment persistence after major cardiovascular events in psoriasis: a retrospective cohort study comparing biologic, systemic, and phototherapy. J Dermatol. 2025;52:1788-95.

25. Lluch-Galcerá JJ, Carrascosa JM, González-Quesada A, Sahuquillo-Torralba A, Rivera-Díaz R, Daudén E, et al. Cardiovascular safety of systemic psoriasis treatments: a prospective cohort study in the BIOBADADERM registry. J Eur Acad Dermatol Venereol. 2025;39:1631-42.

26. Pina Vegas L, Le Corvoisier P, Penso L, Paul M, Sbidian E, Claudepierre P. Risk of major adverse cardiovascular events in patients initiating biologics/apremilast for psoriatic arthritis: a nationwide cohort study. Rheumatology (Oxford). 2022;61:1589-99.

27. Cho H, Kim YJ, Moon IJ, Lee WJ, Won CH, Lee MW, et al. Risk of major adverse cardiovascular events and all-cause mortality among patients with psoriatic disease treated with tumor necrosis factor-α and interleukin-12/23 inhibitors: a nationwide population-based cohort study in Korea. J Dermatolog Treat. 2024;35:2321194.

28. Lee MP, Desai RJ, Jin Y, Brill G, Ogdie A, Kim SC. Association of ustekinumab vs TNF inhibitor therapy with risk of atrial fibrillation and cardiovascular events in patients with psoriasis or psoriatic arthritis. JAMA Dermatol. 2019;155:700-7.

29. Kristensen LE, Strober B, Poddubnyy D, Leung YY, Jo H, Kwok K, et al. Association between baseline cardiovascular risk and incidence rates of major adverse cardiovascular events and malignancies in patients with psoriatic arthritis and psoriasis receiving tofacitinib. Ther Adv Musculoskelet Dis. 2023;15:1759720X221149965.

30. Kridin K, Bieber K, Vorobyev A, Moderegger EL, Olbrich H, Ludwig MA, et al. Biological, as opposed to classic antipsoriatic drug or apremilast, treatment mitigates the risk of death and cardiovascular disease in psoriasis. EBioMedicine. 2025;111:105485.

31. Saurat JH, Stingl G, Dubertret L, Papp K, Langley RG, Ortonne JP, et al. Efficacy and safety results from the randomized controlled comparative study of adalimumab vs. methotrexate vs. placebo in patients with psoriasis (CHAMPION). Br J Dermatol. 2008;158:558-66.

32. Gordon KB, Langley RG, Leonardi C, Toth D, Menter MA, Kang S, et al. Clinical response to adalimumab treatment in patients with moderate to severe psoriasis: double-blind, randomized controlled trial and open-label extension study. J Am Acad Dermatol. 2006;55:598-606.
